# Simulation as a New Tool to Establish Benchmark Outcome Measures in Obstetrics

**DOI:** 10.1371/journal.pone.0131064

**Published:** 2015-06-24

**Authors:** Matt M. Kurrek, Pamela Morgan, Steven Howard, Peter Kranke, Aaron Calhoun, Joshua Hui, Alex Kiss

**Affiliations:** 1 Department of Anesthesia, University of Toronto, Toronto, Ontario, Canada; 2 Department of Anesthesiology, Perioperative and Pain Medicine, Stanford University School of Medicine, Palo Alto, California, United States of America; 3 Department of Anesthesia and Critical Care, University of Wuerzburg, Wuerzburg, Germany; 4 Division of Critical Care, Department of Pediatrics, University of Louisville, Louisville, Kentucky, United States of America; 5 Department of Emergency Medicine, University of California Los Angeles and Olive View-UCLA Medical Center, Los Angeles, California, United States of America; 6 Department of Research Design and Biostatistics, University of Toronto, Toronto, Ontario, Canada; St Francis Hospital, UNITED STATES

## Abstract

**Background:**

There are not enough clinical data from rare critical events to calculate statistics to decide if the management of actual events might be below what could reasonably be expected (i.e. was an outlier).

**Objectives:**

In this project we used simulation to describe the distribution of management times as an approach to decide if the management of a simulated obstetrical crisis scenario could be considered an outlier.

**Design:**

Twelve obstetrical teams managed 4 scenarios that were previously developed. Relevant outcome variables were defined by expert consensus. The distribution of the response times from the teams who performed the respective intervention was graphically displayed and median and quartiles calculated using rank order statistics.

**Results:**

Only 7 of the 12 teams performed chest compressions during the arrest following the ‘cannot intubate/cannot ventilate’ scenario. All other outcome measures were performed by at least 11 of the 12 teams. Calculation of medians and quartiles with 95% CI was possible for all outcomes. Confidence intervals, given the small sample size, were large.

**Conclusion:**

We demonstrated the use of simulation to calculate quantiles for management times of critical event. This approach could assist in deciding if a given performance could be considered normal and also point to aspects of care that seem to pose particular challenges as evidenced by a large number of teams not performing the expected maneuver. However sufficiently large sample sizes (i.e. from a national data base) will be required to calculate acceptable confidence intervals and to establish actual tolerance limits.

## Introduction

Due to the rare occurrence of many critical events, it is usually difficult to gather real life data of sufficient quality and quantity to decide if the management of an actual event fell below what could reasonably be expected of a peer. However, the question whether an intervention has been carried out in a timely manner can be relevant for quality assurance, the design of educational curricula (and also as part of the defense against alleged substandard care).

One approach could be to collect data from realistic crisis simulations and try to define what constitutes an outlier among various performances by plotting the outcome variable in question (for example the time from cardiac arrest to delivery of the neonate, or the time from asystole to chest compression) from a number of different teams. Analogous to the manufacturing industry one could ultimately attempt to set up tolerance limits and define normality accordingly, however, this would require very large sample sizes (i.e. usually several hundred from something like a simulation data registry) [1].

We intended to evaluate the use of simulation data to describe the distribution of management times as an approach to decide if the team management of a simulated obstetrical crisis scenario could be considered an outlier.

## Methods

Institutional ethics review and approval was obtained by Sunnybrook Health Science Centre, University of Toronto Research Ethics Board (Chairperson: Dr. Philip Hebert, 2075 Bayview Ave, Toronto, Ontario M4N 3M5, Canada, approval REB# 351–2006, October 2006) and each participant provided written consent. The subjects of this study were previously reported as part of a project to determine the psychometric properties of a behavioral marking system for obstetrical team training in a high-fidelity simulator [2]. The data have been uploaded to datadryad.org (accession number will be provided during review). The simulation facility consisted of a realistically-equipped hospital room with all relevant equipment available, including anesthesia gas machine, and used the SimMan (Laerdal Medical Canada, Ltd., 51 Nashdene Road #45, Toronto, ON M1V 4C3 Canada) full-scale realistic mannequin with various computer controlled features (voice, anatomically correct airway, heart and breath sounds, etc.). An add-on module was specifically designed and built for the obstetrical scenarios and consisted of a pregnant abdomen with a simulated amniotic sac through which the baby (or babies) had to be delivered via cesarean section. A fetal heart rate simulator provided information on both contractions and fetal heart rate tracings. A pump was installed that could simulate a massive obstetrical hemorrhage.

Four obstetrical simulation scenarios were developed using morbidity and mortality data from the UK Centre for Maternal and Child Enquiries (CMACE). The four scenarios, which had previously been developed included: A) need for Cesarean section under general anesthesia with a difficult airway, can’t intubate/can’t ventilate resulting in hypoxia and leading to pulseless electrical activity; B) severe pre-eclampsia, epidural in situ, non-reassuring fetal heart rate tracing leading to urgent cesarean section and development of pulmonary edema; C) 34-week twin gestation umbilical cord prolapse, emergency cesarean section complicated by amniotic fluid embolism and asystole; D) prolonged fetal bradycardia with emergency cesarean section, occult abruption and massive bleeding.

For the study, 12 multidisciplinary teams (one specialty-certified staff obstetrician, one specialty-certified staff anesthesiologist, 3 staff obstetrical nurses and in some cases, depending on the team’s usual practice pattern, a family doctor), managed all 4 scenarios. The participants’ age (mean±SD) and years in practice (mean±SD) were as follows: nurses (n = 36): 40.4±10.7 and 15.8±11.5; family MDs (n = 6): 37.4±6.4 and 8.6±4.7; obstetricians (n = 12): 47.0±10.0 and 14.5±10.1; anesthesiologists (n = 12): 41.0±7.3 and 7.4±5.5. Each of the teams was recruited from one of the various local teaching or community hospitals and had not received previous high-fidelity simulated obstetrical team training. Every team received a thorough instruction and tour of the simulation centre. At the beginning of each scenario the person to first enter the patient room was given a detailed history of the patient, results of the physical examination with results of pertinent laboratory and the opportunity to interview the patient before scenario begin. Further details were available in the patient’s chart by the bedside. The simulation operator was the same person for all scenarios and the principal investigator oversaw the scenario from the control room with the simulation operator to ensure consistency of presentation. The principal investigator (based on clinical judgment) determined and instructed the simulation operator when to stop the scenario.

Clinically relevant outcome variables for each scenario were defined by a multidisciplinary group of obstetricians, anesthetists and nurses with many years of clinical experience in obstetrics [2].

All sessions were videotaped and time to performance of the outcome measure recorded in seconds by a trained observer who was unaware of the identity of any individual/team.

The times from each team who performed the outcome measure in questions were used to calculate the median and quartiles with their associated 95% confidence intervals. The confidence limits were distribution free and calculated using rank order statistics.

All analyses were carried out using SAS Version 9.2 (SAS Institute, Cary, NC, USA).

## Results

**Fig 1:**
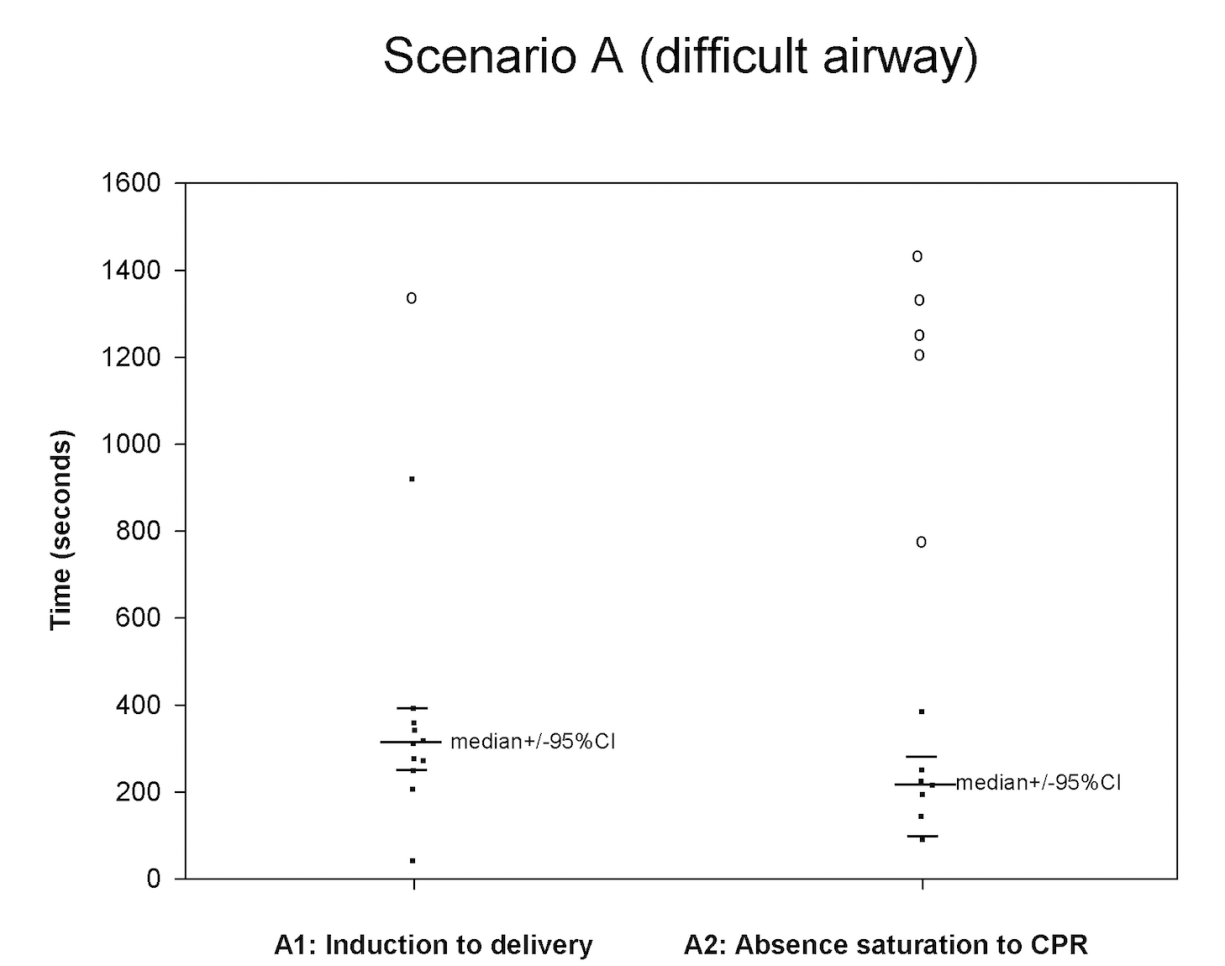
Scenario A. The median was calculated using teams performing the outcome measure (teams not performing the outcome measure are represented by open circles placed at the time when their scenario was stopped).

**Fig 2:**
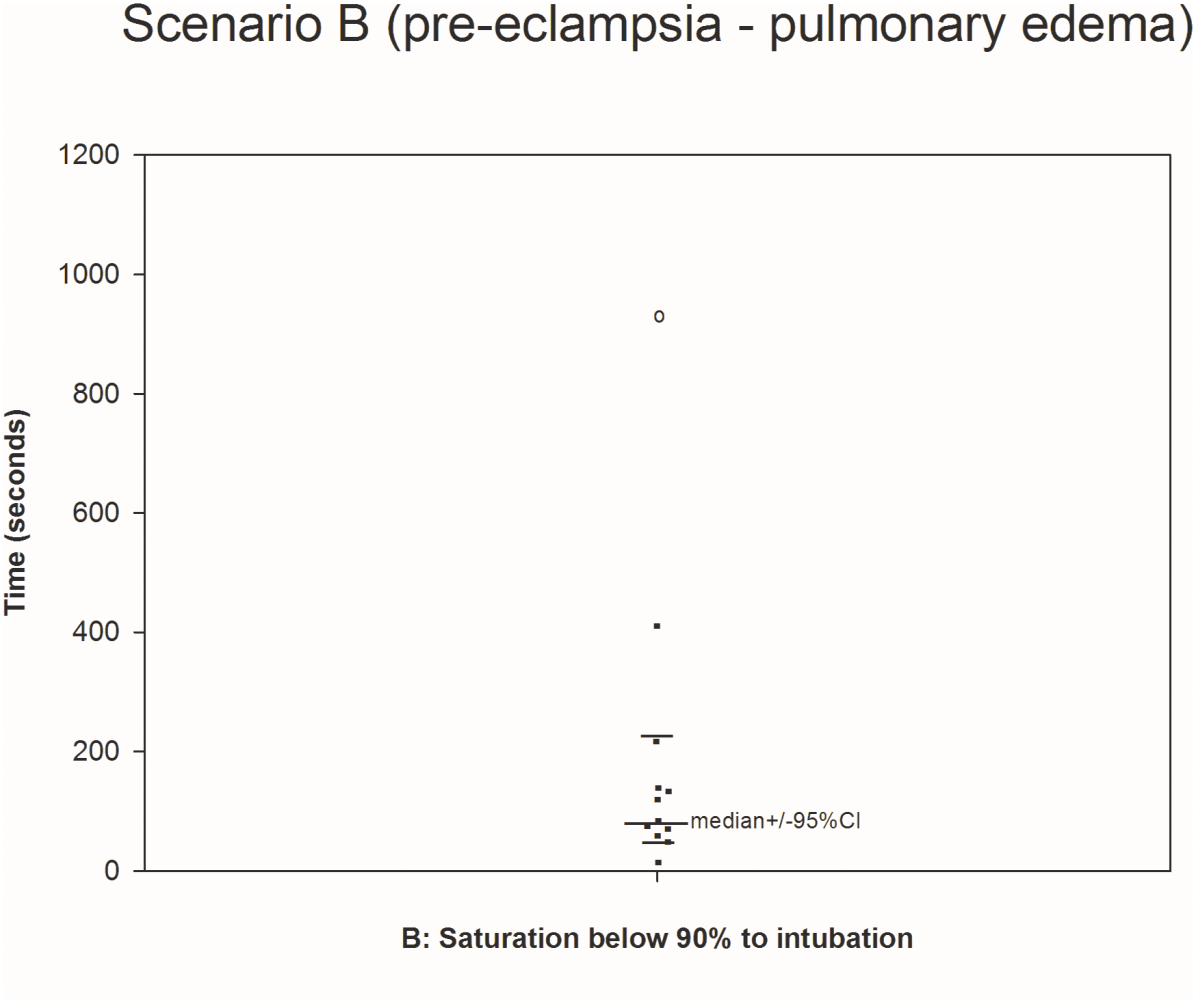
Scenario B. The median was calculated using teams performing the outcome measure (teams not performing the outcome measure are represented by open circles placed at the time when their scenario was stopped).

**Fig 3:**
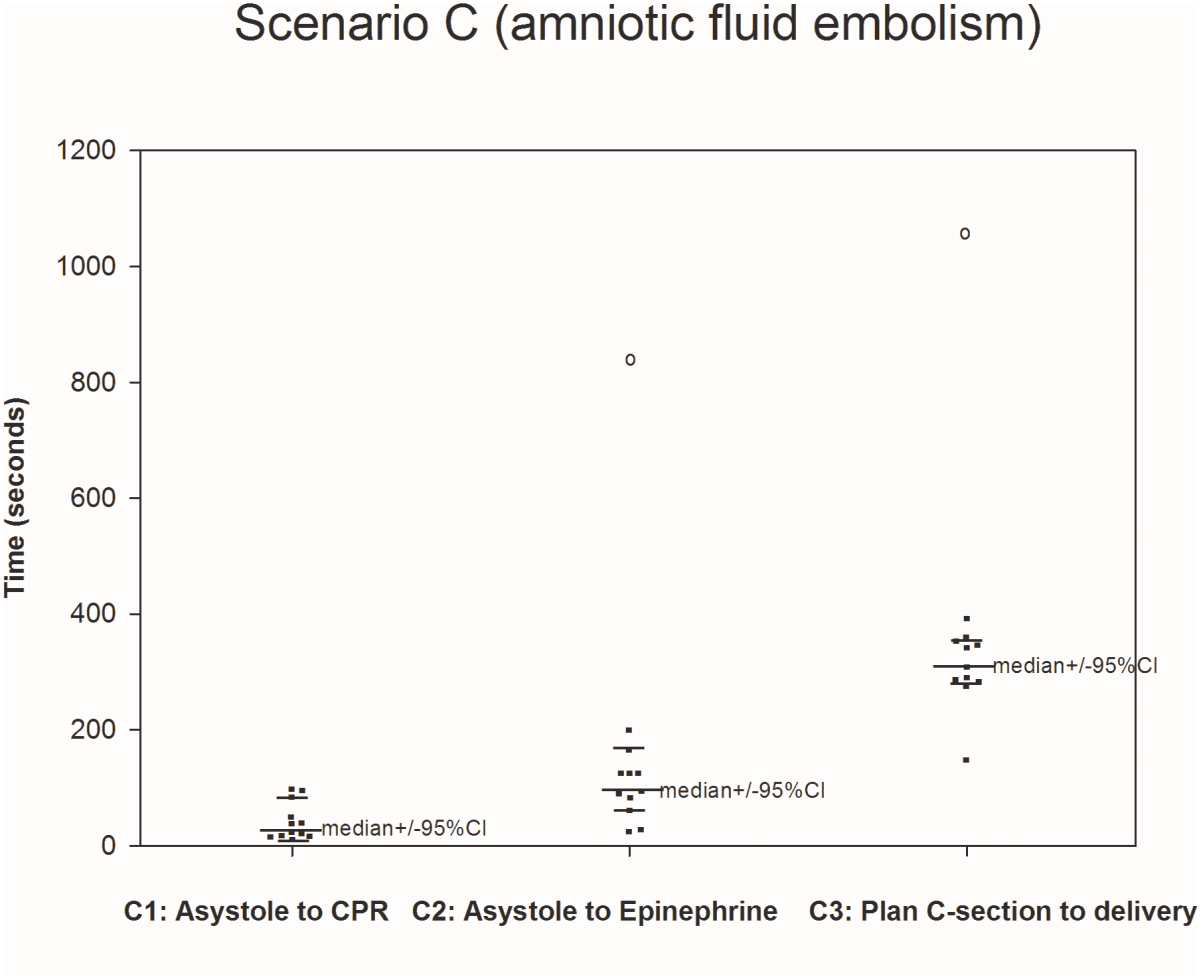
Scenario C. The median was calculated using teams performing the outcome measure (teams not performing the outcome measure are represented by open circles placed at the time when their scenario was stopped).

**Fig 4:**
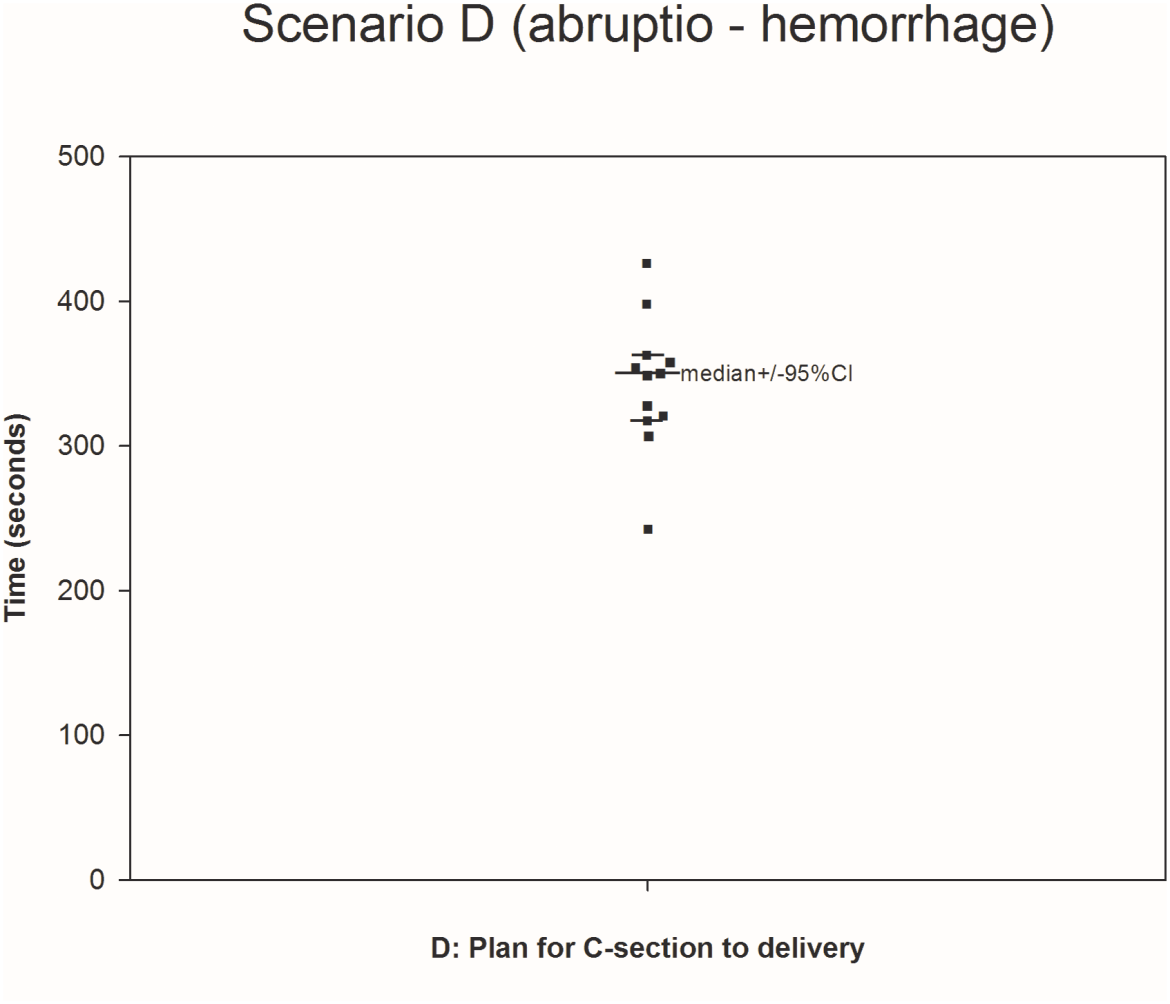
Scenario D. The median was calculated using teams performing the outcome measure (teams not performing the outcome measure are represented by open circles placed at the time when their scenario was stopped).

All twelve teams completed all four scenarios (Figs 1–4.) We were able to calculate the median, 75^th^ and 90^th^ percentiles for time to completion for all 7 clinical outcomes (Table 1) using non-parametric methods to account for non-normally distributed data. The confidence intervals, given the small sample size, were large.

**Table 1:** Outcome measure results.

Scenario Outcome variable (number of teams that performed action)	50^th^ quantile (lower-upper 95% CI in s)	75^th^ quantile (lower-upper 95% CI in s)	90^th^ quantile (lower-upper 95% CI in s)
A1 (11/12)	312s (249–389)	354s (276–917)	389s (354–917)
A2 (7/12)	215s (89–384)	250s (194–384)	384 (221–384)
B (11/12)	79s (57–215)	134s (65–407)	215s (134–407)
C1 (12/12)	28s (14–86)	66s (26–97)	89s (86–97)
C2 (11/12)	93s (63–167)	124s (89–197)	167s (124–197)
C3 (11/12)	304s (275–350)	349s (287–393)	350s (349–393)
D (12/12)	350s (316–361)	359s (350–426)	398s (361–426)

Task C1 (time from onset asystole to initiation of CPR during amniotic fluid embolism) was found to be the shortest one to completion (median 66s [95% CI 26-97s]; 75th quantile 89s [95% CI 86-97s]), whereas task D (time from decision for cesarean section until delivery during massive bleeding) took the longest to complete (median 359s [95% CI 350-426s]; 75th quantile 398s [95% CI 361-426s]).

One outcome variable (Scenario A—cannot intubate, cannot ventilate arrest—outcome A2: time from absence of saturation to initiation of CPR) was only done by 7 of the 12 teams before scenario end.

## Discussion

Time is often one of the few measures that can be extracted from clinical records with at least a moderate degree of certainty following a critical event. There are very few expert opinions on a national level which define expectations (such as the recommendation to perform a perimortem cesarean delivery within 4 minutes of maternal cardiac arrest, as introduced in 1986) [3] and clinicians often have very little objective data to support or refute a claim that an action was so late that it fell below what could reasonably be expected of a peer. We have demonstrated the use rank order statistics to calculate quantiles with confidence limits for management times of critical obstetrical events using data from realistic simulation. This approach could be used to yield information that may assist in the decision if a given performance could be considered normal and could also point to aspects of care that seem to pose particular challenges as evidenced by a large number of teams not performing the expected maneuver.

Ideally, large clinical databases could provide information about the management of critical events, but this is usually not feasible, particularly for events that occur with very low frequency. Realistic simulation, on the other hand, has become very common and allows the exposure of a large number of clinical practitioners to similar critical events in a high fidelity environment [4]. The authors know of only one clinical registry—the recently established ‘Get-with-The-Guidelines Resuscitation’ registry from the American Heart Association—that captures real life cardiac arrest management times (only). The results of such a registry would allow an interesting validation of data from simulated arrests.

Early studies have focused on the use of realistic simulation to evaluate individual technical and non-technical skills [5] whereas more recent works have begun to explore work with entire medical teams [6], recognizing the central role that team performance plays for patient safety. In the context of individual as well as team performance, especially for summative evaluations [7], the question of threshold performance naturally arises: which result is deemed to be acceptable and which result is deemed to be substandard. However establishing credible and accepted cut-off points for performance during summative examinations can be challenging, often relying on subjective expert opinion [8]. The assessment of performance for entire medical teams adds significant challenges that remain to be resolved, such as issues with reliability and relative contributions of individuals versus team skills for overall performance [9]. Most results of individual simulated performances have been validated by comparing different groups of practitioners with various training backgrounds (medical students versus residents versus staff etc.), using scoring templates that are often based on consensus about which action is deemed appropriate (i.e. action ‘x’ must be performed in order to score a point) [10]. The information generated from these scores is therefore limited when trying to evaluate the management of certain critical incidents in real life.

Rather than defining a standard in absolute terms, we attempted to use rank order statistics to calculate quantiles with confidence limits for management times from a small data set of realistic simulations as a possible approach to decide the difference between acceptable and not acceptable, analogous to the concept of defining normality using the Gaussian distribution. This approach of defining normal in a statistical sense is indeed very common for the purpose of creating reference values through tolerance limits especially in manufacturing and laboratory medicine [1,11].

Since the current calculations were done for only 12 teams, the resulting confidence intervals around the calculated median response times and the quantiles were very large. However, with the pooling of results from several simulation centres the amounts of data for these calculations could be significantly expanded and this would provide more acceptable confidence intervals for those parameters as well as make the calculation of tolerance limits possible. A large database of outcomes may also allow to establish a ‘grey zone’ of time-based intervention values from the reference group and thus treat acceptability as a continuous, rather than a binary (acceptable vs. unacceptable) variable.

It may be of interest to create a central data bank of simulation results that could ultimately be used to make those results available to interested users (analogous to an anesthesia registry) and to create reference data for the skills of both teams as well as individual practitioners from various backgrounds during different scenarios. However, if results from several simulations are to be pooled, then it will be necessary to ensure that the scripting of the scenarios is comparable.

It has to be remembered that the tolerance limits from such data would be derived from realistic simulations and this should of course be kept in mind when trying to make inferences on clinical performance. While the realism of case presentations and simulated incidents is generally rated as fairly high among participants [12], most participants anticipate that something will happen during the simulation and may thus display heightened vigilance and faster response times when compared to real life. Also, especially with respect to invasive procedures (including cesarean sections) the threshold to perform such an intervention and the time to complete the procedure may not necessarily be the same as in real life.

While time is often an easily accessible measure, it may not always be a good indicator of competent or even optimal performance. Time metrics may work well when individuals or teams are presented with situations in which single diagnoses and/or treatments are equally plausible at the beginning. When there are multiple possibilities and the "real one" isn't known until more information is collected, practitioners who—just by “lucky guessing”—will have a short response time even though their decision-making or problem solving may not be ideal. On the other hand, someone who choses something equally valid at first would be assigned a longer response time (and ‘worse’ performance) even though they both made an equally good first choice and the longer response time may indeed reflect a high degree of diligence. The most appropriate metric for those latter scenarios, taking in account decision-making and problem solving as markers of quality performance may be a non-technical marker (perhaps in addition to time) and will have to be decided for each scenario in question.

Furthermore, there are likely variations in performance that will lead a clinician to a good outcome and vice versa. The “standard of care” defined as what a reasonably prudent medical provider would or would not have done under the same of similar circumstances. Currently, little is known about how a reasonable and prudent practitioner performs their work. Simulation might allow a window (though not perfect) into this very important area.

An outcome measure that is not performed by a significantly large number of participants before the end of the scenario may reveal particularly challenging clinical encounters for practitioners. While the commencement of CPR after the loss of the saturation signal (indicating a pulseless electrical activity arrest following a cannot intubate/cannot ventilate condition) was considered an essential action and tracked as part of the study, it was not done by 41% of the participating teams. This was surprising and while such a finding could result from a lack of realism of the scenario, the participants in our study did not indicate that they found the scenario unrealistic. If this is consistently observed in similar simulations, then this type of observation is extremely important because it would allow planning educational interventions in order to address what may be perceived as shortcomings in clinical management.

## Conclusion

In summary, we have shown the use of rank order statistics to calculate quantiles with confidence limits for management times of critical obstetrical events using data from realistic simulation. This approach could be used to describe the distribution of treatment times in order to assist in deciding what performance may constitute an outlier. It can also identify particular challenges of clinical practice and allow the development of educational curricula. While the information derived from simulation has to be interpreted with a high degree of caution for a clinical context, it may represent a further ‘added value’ of important step in establishing simulation as a training tool and to provide information that could be used in appropriate clinical context for adverse events. Large amounts of data (such as from a simulation registry) would allow calculating acceptable confidence intervals for the required outcome parameters as well as actual tolerance limits.
